# New and Simple Endo-extra Oesophagotracheal Method of Developing the Fistula for the Implantation of a Voice Prosthesis

**DOI:** 10.1155/DTE.3.189

**Published:** 1997

**Authors:** G. Lichtenberger

**Affiliations:** Department of ORL Head and Neck Surgery Szent Rókus Hospital Gyulai Pál u. 2 Budapest H-1085 Hungary

## Abstract

The author has simplified the Blom-Singer^1^ puncture. The fistula is formed with the help of his new device, the endo-extralaryngeal needle carrier and pointed metal cone fixed to a catheter. The voice prosthesis is placed in the fistula formed thus in an endo-extra oesophagotracheal way. The advantage of the method is, that when the fistula being formed from inside out, the back wall of the oesophagus will not become injured.


*Diagnostic and Therapeutic Endoscopy*, 1997, Vol. 3, pp. 189–191
Reprints available directly from the publisher
Photocopying permitted by license only

© 1997 OPA (Overseas Publishers Association)
Amsterdam B.V. Published in The Netherlands
by Harwood Academic Publishers
Printed in Singapore

### New and Simple Endo-extra Oesophagotracheal Method of Developing the Fistula for the Implantation of a Voice Prosthesis

G. LICHTENBERGER

*Department of ORL, Head and Neck Surgery, Szent Rókus Hospital, Gyulai Pál u. 2, H-1085 Budapest, Hungary*

*(Received 25 June 1996; in final form 17 September 1996)*

**The author has simplified the Blom-Singer[1] puncture. The fistula is formed with the help of his new device, the endo-extralaryngeal needle carrier and pointed metal cone fixed to a catheter. The voice prosthesis is placed in the fistula formed thus in an endo-extra oesophagotracheal way. The advantage of the method is, that when the fistula being formed from inside out, the back wall of the oesophagus will not become injured.**

*Keywords:* Endo-extralaryngeal needle carrier, implantation, JET anesthesia, voice prosthesis

### INTRODUCTION

The Blom-Singer[1] puncture and the implantation of the voice prosthesis have proved to be a great advantage to laryngectomized patients who can not learn oesophagopharyngeal voice production and are unable or unwilling to speak with the electro-larynx.

The puncture and the implantation is often performed simultaneously with laryngectomy. In our opinion, it is better to carry out a secondary puncture and voice prosthesis implantation. The Blom-Singer[1] puncture and the implantation of the voice prosthesis opened new possibilities in the treatment of laryngectomized patients. However the formation of the secondary fistula was – besides involving certain risk – rather complicated and done under local anaesthesia it proved to be unpleasant or even painful for the patient.

By using a Haslinger oesophagoscope the back wall of the oesophagus and of the pharynx may be injured if we cannot hit the opening of the oesophagoscope with the bent fistula forming trocar. The same injury may be caused when the tube, having been led in, is turned by 180° so that its bevelled end could protect the back wall of the oesophagus.

---

Correspondence: György Lichtenberger, MD, PhD., Professor and Chairman, Dept. of ORL, Head and Neck Surgery, Szent Rókus Hospital, Gyulai Pál u. 2, H-1085 Budapest, Hungary. Tel: (36-1) 266-1466; Fax: (36-1) 266-2768.

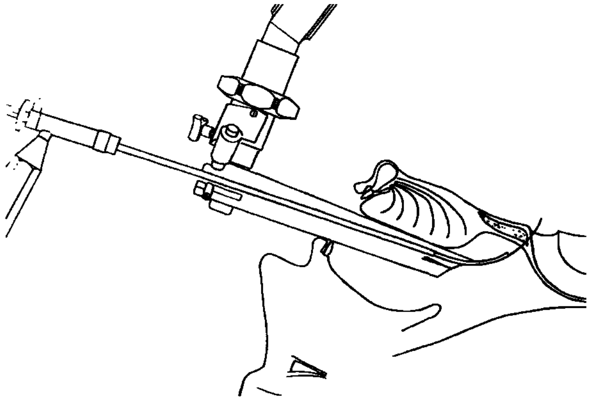

FIGURE 1 The needle with the thread is pushed through in the upper third of the tracheostoma.

2A

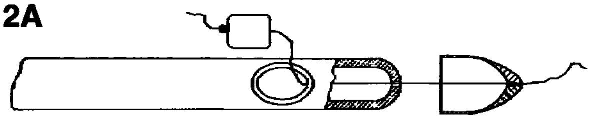

FIGURE 2A The thread is pushed through a counterfixing pierced ball and then knotted.

2B

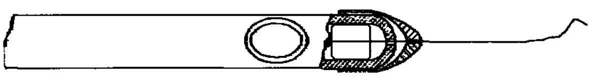

FIGURE 2B The distal end of the catheter is to be seen with the thread, fixed between the pointed metal cone and the counterfixing ball.

The injury may also be brought about when using oesophagoscope, where the opening for the bent trocar is formed at 1–2 cm proximally to the distal opening of the tube of the oesophagoscope.

### MATERIALS AND METHODS

The method I have developed is the following:

The operation is performed under JET anesthesia leaving enough room for the surgeon in the area of the stoma.

The pharynx is opened with the laryngoscope that is then led up to the entrance of the oesophagus. Through the laryngoscope the distal end of the endo-extralaryngeal needle carrier* is led into the oesophagus. The distal end of the device can be seen up to the point where the oesophagus opens. Now the distal bent part of the instrument can be touched from the outside. The instrument is pushed forward as long as its distal bent end is palpable in the upper third of the tracheostoma. The needle with the thread (Prolen 2/0) is pushed through in the upper third of the tracheostoma (Fig. 1).

In the meantime the next instrument (to be seen in Fig. 2A and 2B) will be prepared by the assistant: he pushes a strong (PDS) thread through the opening of the metal cone.

The thread is then led with the needle inside the distal part of the 18 Ch catheter and led outside through one of the side holes of the plastic catheter. Here the thread is pushed through a counterfixing pierced ball and then knotted (Fig. 2A). In the next step the counterfixing piece is put back into the catheter, that results the condition seen in Fig. 2B. By pulling the thread the end of the catheter is fixed between the metal cone and the counterfixing piece. Then the end of the thin thread (Prolen 2/0) hanging from the patient's mouth is fastened with the strong (PDS) one. With the help of the thin thread the strong thread is pulled through the stitching canal and is pulled on a

*The endo-extralaryngeal needle carrier instrument is available at the R. Wolf Ltd., D-75438 Knittlingen (Germany) Pforzheimer Str. 32.

little. By pulling it, the pointed end of the metal cone perforates the soft parts and pulls the catheter with itself.

In certain cases – when the metal cone can already be seen – a 1 mm long cut with a scalpel will be needed on the above laying soft parts. This cut makes the easy pulling of the catheter possible.

After the fistula has been thus prepared for the implantation of the prosthesis, the metal cone, the thread and the conterfixing piece are cut off the end of the catheter. The PROVOX spit is then led into the catheter and pulled out with it through the mouth. The voice prosthesis will be fixed on to the spit, then it is pulled into the opening of the fistula and it will be fixed there.

When implantation is finished the state of the prosthesis will be controlled with the oesophagoscope also from the inside.

### RESULTS

We have performed the above operation in three patients, following the insufflation test. All the three patients were able to speak continuously and understandably after the implantation.

### CONCLUSION

This method of forming the fistula in the endo-extra oesophagotracheal way is simple and safe to apply. The risk of injuring the back wall of the pharynx with the trocar used in Blom-Singer puncture method is eliminated.

The use of JET anaesthetic ensures complete freedom from pain and leaves enough room for the surgeon in the area of the stoma while operating.

### *Reference*

[1] Blom, E.D. and Singer, M.I.: Surgical prosthetic approaches for post-laryngectomy voice restoration. In: Keith, R.L. and Darley, F.L. (eds.): *Laryngectomy rehabilitation*. Houston: College Hill Press 1979: 251–276.

